# Challenges and advances in the management of post-transplant lymphoproliferative disease

**DOI:** 10.1590/2175-8239-JBN-2024-E012en

**Published:** 2024-11-22

**Authors:** Tainá Veras de Sandes-Freitas, Raoni de Oliveira Domingues-da-Silva, Fernando Barroso Duarte

**Affiliations:** 1 Universidade Federal do Ceará, Complexo Hospitalar, Empresa Brasileira de Serviços Hospitalares (Ebserh), Fortaleza, CE, Brazil.; 2 Universidade Federal do Ceará, Faculdade de Medicina, Fortaleza, CE, Brazil.

Compared to the general population, recipients of solid organ transplants (SOT) and hematopoietic stem cell transplants (HSCT) face an up to 40-fold increased risk of developing lymphoproliferative disorders, malignant neoplasms characterized by the monoclonal or polyclonal proliferation of lymphoid cells^1^. In the context of transplantation, these disorders are known as post-transplant lymphoproliferative disease (PTLD), which is associated with substantial morbidity and mortality.

In this edition of BJN, Francisco et al.^2^ present the findings from a cohort study involving 41 kidney transplant recipients diagnosed with PTLD and concurrent Epstein-Barr virus (EBV) viremia. Most cases were diagnosed late post-transplantation (median of 8.6 years), 80.5% were monomorphic PTLD, and 61% were classified as Lugano stage IV. Over half of the patients died within two years, underscoring the severity of the disease. Mortality risk factors included older age, Lugano stage IV (disseminated extranodal involvement), and higher EBV viral load at diagnosis^2^.

Classically, EBV-related PTLD occurs early after transplantation and is often linked to primary infection in recipients with an EBV serological mismatch to the donor (EBV IgG-positive donor and EBV IgG-negative recipient), frequently involving the graft. Late-onset cases, increasingly reported in international cohorts, show a weaker association with EBV and are more commonly related to older age and prolonged exposure to immunosuppressive therapies. These cases tend to involve multiple extranodal sites^3^.

In the cohort described by Francisco et al.^2^, the presence of the virus in tumor cells was not reported, despite the association with positive EBV viremia, making it impossible to definitively classify the PTLD as EBV-related. The relationship between viremia and viral detection in tissue (via immunohistochemistry or, ideally, in situ hybridization) is not obligatory. Notably, EBV viremia may serve as a marker for excessive immunosuppression, consistent with the state of an individual with an active neoplasm^4^. Regardless of whether PTLD is EBV-related, viremia was identified as an independent risk factor for mortality, which is a key finding of this study.

Given the association between PTLD and primary EBV infection, international guidelines recommend monitoring EBV viremia in EBV-seronegative recipients of organs from EBV-seropositive donors, particularly during the first year post-transplant or periods of intensified immunosuppression. Viremia screening may also be indicated regardless of serological status in SOT recipients undergoing more intense immunosuppression, such as those receiving intestinal or lung transplants, and in HSCT recipients. Although robust evidence is limited, guidelines recommend reducing immunosuppression in patients with rising viral loads. Other preemptive strategies discussed in the literature include antivirals, intravenous immunoglobulin (IVIG), and rituximab^5,6,7^. However, it is essential to note that no definitive viral load threshold has been established to trigger preemptive treatment.

The diagnosis of late-onset PTLD, as reported in the study, is particularly challenging due to its highly variable clinical presentation, which depends on the site of involvement. The first-line intervention for managing established PTLD is the reduction of maintenance immunosuppression. Additional treatments such as rituximab, chemotherapy (CHOP regimen: cyclophosphamide, doxorubicin, vincristine, and prednisone), radiotherapy, and surgery may be required, depending on the tumor’s location and histological subtype. Determining the tumor’s immunohistochemical profile is crucial for guiding therapeutic decisions and assessing prognosis^8^.

Since complete cessation of maintenance immunosuppression carries the risk of acute and chronic rejection, mammalian target of rapamycin (mTOR) inhibitors have emerged as a therapeutic strategy for selected patients. This approach aims to leverage both the antineoplastic and immunosuppressive properties of mTOR inhibitors, particularly in patients with EBV-associated PTLD^9^.

More recently, novel therapies have been evaluated for the management of PTLD, offering new prognostic perspectives for these patients. These treatments include proteasome inhibitors, BTK inhibitors, PI3K/Akt/mTOR pathway inhibitors, BCL-2 inhibitors, hypomethylating agents, checkpoint inhibitors (anti-PD-1), EBV-specific T-cell therapies, CAR-T cell therapy, and even autologous hematopoietic stem cell transplantation^10^.
