# Supplementary material for: Challenges and advances in the management of post-transplant lymphoproliferative disease
Source: J Bras Nefrol. 2024 Nov 22;46(4):e2024E012. doi: 10.1590/2175-8239-JBN-2024-E012en (PMC11588344; doi:10.1590/2175-8239-JBN-2024-E012en)
Supplement: Translation

# Translation: Desafios e avanços no manejo da doença linfoproliferativa pós-transplante

**DOI:** 10.1590/2175-8239-JBN-2024-E012pt

**Authors:** Tainá Veras de Sandes-Freitas, Raoni de Oliveira Domingues-da-Silva, Fernando Barroso Duarte

**Affiliations:** 1 Universidade Federal do Ceará, Complexo Hospitalar, Empresa Brasileira de Serviços Hospitalares (Ebserh), Fortaleza, CE, Brasil.; 2 Universidade Federal do Ceará, Faculdade de Medicina, Fortaleza, CE, Brasil.

Receptores de transplantes de órgãos sólidos (TOS) e de células-tronco hematopoiéticas (TCTH) apresentam um risco até 40 vezes maior que a população geral de desenvolver desordens linfoproliferativas, ou seja, neoplasias malignas caracterizadas por proliferação mono ou policlonal das células linfoides^1^. No contexto do transplante, essas desordens são conhecidas como doença linfoproliferativa pós-transplante (DLPT), uma condição de elevada morbimortalidade.

Nesta edição do BJN, Francisco et al.^2^ apresentaram os resultados de uma coorte de 41 receptores de transplante renal com DLPT e concomitante viremia para o vírus Epstein-Barr (EBV). Os casos foram, em sua maioria, diagnosticados tardiamente após o transplante (mediana de 8,6 anos), eram DLPT monomórficas (80,5%), 61% classificados como Lugano IV e mais da metade dos pacientes faleceu ao longo de 2 anos, demonstrando a gravidade da doença. Os fatores de risco para o óbito foram idade mais avançada, estágio Lugano IV (envolvimento extranodal disseminado) e maior carga viral para o EBV ao diagnóstico^2^.

Classicamente, a DLPT relacionada ao EBV ocorre mais precocemente após o transplante, estando frequentemente associada à primo-infecção em receptores de transplante com *mismatch* sorológico com o doador (doador EBV IgG positivo e receptor EBV IgG negativo) e, não raramente, envolve o enxerto. Casos tardios, que têm sido cada vez mais frequentemente reportados nas coortes internacionais, têm associação menos clara com o vírus EBV, estando associados à idade mais avançada e, principalmente, à longa exposição aos fármacos imunossupressores, e costuma envolver diferentes sítios extranodais^3^.

Na coorte descrita por Francisco et al.^2^, apesar da associação com viremia positiva para EBV, a positividade do vírus nas células tumorais não foi reportada, não sendo possível afirmar tratar-se de DLPT relacionada ao EBV, dado que a associação entre viremia e evidência do vírus no tecido (detectado por imuno-histoquímica ou, idealmente, hibridização *in situ*) não é obrigatória. Digno de nota, viremia por EBV pode ser um marcador de um estado de excesso de imunossupressão, condizente com a condição de um indivíduo com neoplasia em atividade^4^. De toda forma, independentemente de tratar-se de DLPT relacionada ou não ao EBV, a viremia foi um fator de risco independente para mortalidade, relevante achado deste estudo.

Dada a associação da DLPT com a primo-infecção pelo EBV, as diretrizes internacionais recomendam monitoramento da viremia para EBV em receptores EBV-soronegativos que receberam órgãos de doadores EBV-soropositivos durante o primeiro ano após o transplante ou durante períodos de intensificação da imunossupressão. O rastreio da viremia pode também ser indicado, independentemente do status sorológico em receptores de TOS submetidos a imunossupressão mais intensa, como intestino e pulmão, e no TCTH. Apesar da escassez de evidências robustas, as diretrizes sugerem redução da imunossupressão para pacientes com aumento da carga viral. Outras estratégias preemptivas discutidas na literatura são o uso de antivirais, imunoglobulina intravenosa (IVIG) e rituximabe^5–7^. Vale ressaltar que não há evidências que estabeleçam um ponto de corte de viremia que enseje o tratamento preemptivo.

O diagnóstico em casos de PTLD tardio, como os reportados no estudo em questão, é mais desafiador, pois a apresentação clínica é bastante variável a depender do sítio de acometimento. Para o manejo da DLPT instalada, a intervenção de primeira linha é a redução da imunossupressão de manutenção. Rituximabe, ciclos de quimioterapia baseados em ciclofosfamida, doxorrubicina, vincristina e prednisona (CHOP), radioterapia e cirurgia podem ser necessários, a depender da localização e do padrão histológico. A determinação do perfil imuno-histoquímico do tecido tumoral é fundamental para decisão terapêutica e para avaliação prognóstica^8^.

Tendo em vista que a completa suspensão da imunossupressão de manutenção pode colocar o paciente sob maior risco de rejeição aguda e crônica, o uso dos inibidores da *mammalian target of rapamycin* (mTOR) têm sido uma estratégia para pacientes selecionados em que se planeja combinar efeito antineoplásico e efeito imunossupressor, principalmente para pacientes com DLPT associada ao EBV^9^.

Mais recentemente, novas terapias têm sido avaliadas para o manejo da DLPT, trazendo novas perspectivas de prognóstico para esses pacientes. Entre esses tratamentos, destacam-se os inibidores de proteassoma, inibidores de BTK, inibidores da via PI3K/Akt/mTOR, inibidores BCL-2, agentes hipometilantes, inibidores de *checkpoint* (anti-PD-1), terapias com células T específicas para EBV, terapia com células CAR-T e mesmo o transplante autólogo de células-tronco hematopoéticas^10^.
